# Amphibian species traits, evolutionary history and environment predict *Batrachochytrium dendrobatidis* infection patterns, but not extinction risk

**DOI:** 10.1111/eva.12520

**Published:** 2017-09-03

**Authors:** Dan A. Greenberg, Wendy J. Palen, Arne Ø. Mooers

**Affiliations:** ^1^ Department of Biological Sciences and Crawford Laboratory of Evolutionary Studies Simon Fraser University Burnaby BC Canada; ^2^ Department of Biological Sciences and Earth to Ocean Research Group Simon Fraser University Burnaby BC Canada

**Keywords:** amphibian, *Batrachochytrium dendrobatidis*, chytridiomycosis, extinction, phylogeny, resistance, tolerance, traits

## Abstract

The fungal pathogen *Batrachochytrium dendrobatidis* (*B. dendrobatidis*) has emerged as a major agent of amphibian extinction, requiring conservation intervention for many susceptible species. Identifying susceptible species is challenging, but many aspects of species biology are predicted to influence the evolution of host resistance, tolerance, or avoidance strategies towards disease. In turn, we may expect species exhibiting these distinct strategies to differ in their ability to survive epizootic disease outbreaks. Here, we test for phylogenetic and trait‐based patterns of *B. dendrobatidis* infection risk and infection intensity among 302 amphibian species by compiling a global data set of *B. dendrobatidis* infection surveys across 95 sites. We then use best‐fit models that associate traits, taxonomy and environment with *B. dendrobatidis* infection risk and intensity to predict host disease mitigation strategies (tolerance, resistance, avoidance) for 122 Neotropical amphibian species that experienced epizootic *B. dendrobatidis* outbreaks, and noted species persistence or extinction from these events. Aspects of amphibian species life history, habitat use and climatic niche were consistently linked to variation in *B. dendrobatidis* infection patterns across sites around the world. However, predicted *B. dendrobatidis* infection risk and intensity based on site environment and species traits did not reveal a consistent pattern between the predicted host disease mitigation strategy and extinction outcome. This suggests that either tolerant or resistant species may have no advantage in ameliorating disease during epizootic events, or that other factors drive the persistence of amphibian populations during chytridiomycosis outbreaks. These results suggest that using a trait‐based approach may allow us to identify species with resistance or tolerance to endemic *B. dendrobatidis* infections, but that this approach may be insufficient to ultimately identify species at risk of extinction from epizootics.

## 1 INTRODUCTION

Identifying the most at‐risk species in a community has emerged as a key goal in conservation to direct limited funding to those species most in need (Bottrill et al., 2008). Comparative analyses that can link aspects of species biology and exposure to threats can potentially be used to predict risk for data‐poor species and understudied regions (Bland, Collen, Orme, & Bielby, 2015; Cardillo & Meijaard, 2012). These analyses can then be used to identify species that are susceptible to population declines and so that are most likely to benefit from management intervention.

Amphibians are one of the most threatened vertebrate classes, with thousands of species at risk of extinction (Hoffmann et al., 2010; Stuart et al., 2004). A major driver of amphibian extinction is the disease chytridiomycosis, caused by the fungal pathogen *Batrachochytrium dendrobatidis* (*B. dendrobatidis*) (Berger et al., 1998; Stuart et al., 2004). Over a hundred amphibian species may have already been driven to extinction by this disease (Skerratt et al., 2007), and *B. dendrobatidis* ‐induced declines continue across the world (Catenazzi, Lehr, Rodriguez, & Vredenburg, 2011; Hirschfeld et al., 2016; Woodhams et al., 2008). There is concern that future extinctions may occur through the spread of different *B. dendrobatidis* strains, particularly virulent strains to naïve regions (Fisher & Garner, 2007; Kolby et al., 2015; Skerratt et al., 2007), or through the emergence of new fungal pathogens (Martel et al., 2014). Not all species are equally susceptible to *B. dendrobatidis* infection or the disease chytridiomycosis (Gahl, Longcore, & Houlahan, 2012; Searle et al., 2011), and patterns of both *B. dendrobatidis* infection and associated population declines vary widely among taxonomic groups (Baláž et al., 2014; Bancroft et al., 2011; Stuart et al., 2004). Therefore, understanding both the mechanistic basis of susceptibility and how to identify susceptible species *a priori* is a key question for proactive management strategies such as establishing amphibian ex situ holdings before *B. dendrobatidis* spreads to the few remaining naïve regions (Mendelson et al., 2006; Olson et al., 2013).

The disease susceptibility of any host is the product of its ability to avoid exposure to the pathogen, its ability to resist infection when pathogens are encountered, and its tolerance of the pathological effects of infection (Medzhitov, Schneider, & Soares, 2012). These axes of disease susceptibility are recognized as representing distinct evolutionary strategies to cope with disease by either limiting potential infections or offsetting the fitness consequences of infections, with presumed trade‐offs associated with each strategy (Råberg, Sim, & Read, 2007; Roy & Kirchner, 2000). In turn, these different strategies predict different patterns of pathogen infection risk (i.e., the probability of both encountering and becoming infected by a pathogen) and intensity (i.e., the pathogen load) for sympatric hosts within an infected community (Roy & Kirchner, 2000). Avoidant species are those that minimize their risk of pathogen contact, primarily through behaviours that may limit overlap between host and pathogen in the environment (e.g., avoidance of water; Rowley & Alford, 2007). Species adopting an avoidance strategy should have a relatively low risk of infection due to low pathogen encounter rates, but when infected suffer a high infection intensity due to a lack of resistance mechanisms. If pathogen encounter rates are high, and largely unavoidable, then selection may favour alternative tactics based on resistance or tolerance mechanisms (Donnelly, White, & Boots, 2015; Roy & Kirchner, 2000). Resistant species could have high or low infection risk (depending on their ability to resist initial infection), but generally exhibit comparatively low intensity infections. Tolerant species are expected to exhibit both high infection risk and high infection intensity, but unexpectedly low mortality. Finally, species with both low infection risk and intensity could exhibit either an avoidance or resistance strategy. Species fitting this profile could also possess elements of both strategies by retaining resistance mechanisms while avoiding the pathogen, but this strategy is likely to be rare given expected trade‐offs. It is generally expected that highly tolerant or resistant species are typically less susceptible to population‐level decline associated with emerging infectious diseases (Reeder, Pessier, & Vredenburg, 2012; Woodhams et al., 2006), and therefore, these species would be of low priority for proactive conservation management.

**Figure 1:**
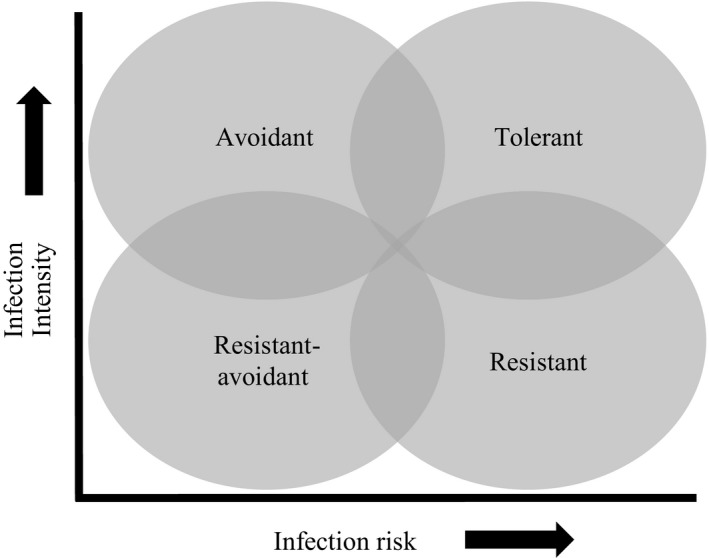
Conceptual outline for predicted patterns of pathogen infection risk and intensity for different host strategies to manage disease. Tolerant hosts would be characterized by a high risk and high infection intensity, as these hosts invest in ameliorating the fitness costs of infection. Resistant hosts are expected to have higher risk, but low levels of infection. Avoidant hosts will have low infection risk, but when infected, suffer a high infection intensity due to a lack of resistance mechanisms. Finally, a host with both lower risk and lower infection intensity could be resistant, avoidant, or both

The disease outcome for any infected host is dictated by the interactions between host susceptibility, pathogen virulence and their environmental context (James et al., 2015). This complex interaction can confound our ability to understand why certain species suffer a higher pathogen burden when comparing across distinct pathogen strains and geographic locations. However, examining patterns of relative infection risk and intensity across hosts from the same infected community, and therefore experiencing the same pathogen and environmental conditions, may allow us to identify patterns of infection consistent with these evolutionary disease mitigation strategies (Figure 1). If we assume that hosts in these infected communities have the same potential exposure to the pathogen, then differences in infection prevalence across hosts can be attributed to differences in infection risk and differences in infection intensity can reflect their ability to resist or tolerate the pathogen when infected. Comparing infection patterns across hosts, sampled in the same space and time, provides the opportunity to test whether aspects of hosts' biology may be associated with a particular disease mitigation strategy.

It has been established that some amphibian species appear to be more tolerant to *B. dendrobatidis* infection (Reeder et al., 2012; Searle et al., 2011), while others possess various mechanisms to resist infection (Ellison et al., 2015; Eskew, Worth, Foley, & Todd, 2015), and differences in species ability to avoid infection can arise from their ecology and behaviour (Burrowes, Martes, Torres‐Ríos, & Longo, 2017; Richards‐Zawacki, 2010; Rowley & Alford, 2007). A diverse set of traits appear to influence these disease mitigation strategies in amphibians, including immune system defences (Ellison et al., 2015; Savage & Zamudio, 2011), antimicrobrial peptides (Woodhams et al., 2006) and skin microbiota (Harris, James, Lauer, Simon, & Patel, 2006), which have all been linked to variation in susceptibility to chytridiomycosis. Many other aspects of species biology, including their life history, ecology, behaviour or physiology, also have the potential to influence the evolution of avoidance, resistance and tolerance strategies (Han, Schmidt, Bowden, & Drake, 2015; Johnson et al., 2012; Nowakowski et al., 2016). Although ecological traits offer a less direct, mechanistic link to avoidance, resistance and tolerance, data for these traits are more readily available for a broad range of species compared to the few species that have been directly assessed for *B. dendrobatidis* susceptibility (Ellison et al., 2015; Gahl et al., 2012; Searle et al., 2011). If particular biological traits are consistently associated with avoidance, resistance or tolerance strategies, then this may provide a shortcut to identify susceptible species in amphibian assemblages at risk of *B. dendrobatidis* outbreaks.

Patterns of population declines and local extinctions among species following *B. dendrobatidis* epizootics have given us some insight into the particular traits that appear to confer susceptibility to chytridiomycosis (Bielby, Cooper, Cunningham, Garner, & Purvis, 2008; Lips, Reeve, & Witters, 2003; Smith, Lips, & Chase, 2009). Traits associated with species decline due to chytridiomycosis include aquatic habitats, small geographic range size, large body size and low fecundity (Bielby et al., 2008; Lips et al., 2003). Physiology has emerged as another important component of infection risk, as species with high thermal tolerance can both preferentially select higher temperatures to help clear infections (a resistance mechanism) or reduce their exposure to the pathogens by occupying microhabitats outside the thermal growth range of *B. dendrobatidis* (an avoidance mechanism) (Catenazzi, Lehr, & Vredenburg, 2014; Nowakowski et al., 2016; Richards‐Zawacki, 2010). A species overall exposure to *B. dendrobatidis* in its environment may also subsume any effects of host biology on patterns of both infection and population decline (Murray & Skerratt, 2012; Murray et al., 2011), and therefore, exposure is important to account for given that *B. dendrobatidis* infection risk can vary considerably across environments (Liu, Rohr, & Li, 2012). Evolutionary history may be related to *B. dendrobatidis* ‐induced extinctions, with conflicting evidence suggesting that *B. dendrobatidis* epizootics either homogenize amphibian diversity (Smith et al., 2009), or alternatively cause phylogenetically random extinctions (Crawford, Lips, & Bermingham, 2010). This suggests that a combination of species traits and evolutionary history, when accounting for exposure and environmental conditions, may allow us to identify evolutionary strategies of avoidance, resistance and tolerance among species, and link these strategies to risk of extinction from chytridiomycosis epizootics.

There have been no direct tests of whether tolerance, resistance or avoidance strategies are correlated with differential survival from chytridiomycosis epizootics across species, but we can derive some predictions from both observation and theory. It has been observed that tolerant species can persist in infected sites despite the extinction of more susceptible species (Reeder et al., 2012; Retallick, McCallum, & Speare, 2004), suggesting that tolerant species may have a higher survival probability during chytridiomycosis epizootics. For resistant species, survival may depend on whether hosts can sustain the fitness costs of resistance when infection rates and, subsequently infection intensity, increase during epizootic events (Briggs, Knapp, & Vredenburg, 2010; Roy & Kirchner, 2000), or if these heightened rates of transmission may overwhelm host defences leading to mortality. We may expect that avoidant species may disproportionately suffer during epizootics due to low investment in resistance or tolerance mechanisms (Medzhitov et al., 2012); however, if these avoidant hosts can consistently limit exposure, this may increase their probability of survival relative to resistant or tolerant species. Understanding how extinction risk varies across these distinct disease strategies is critical if we aim to manage species based on their ability to resist, tolerate or avoid *B. dendrobatidis* infections.

**Figure 2:**
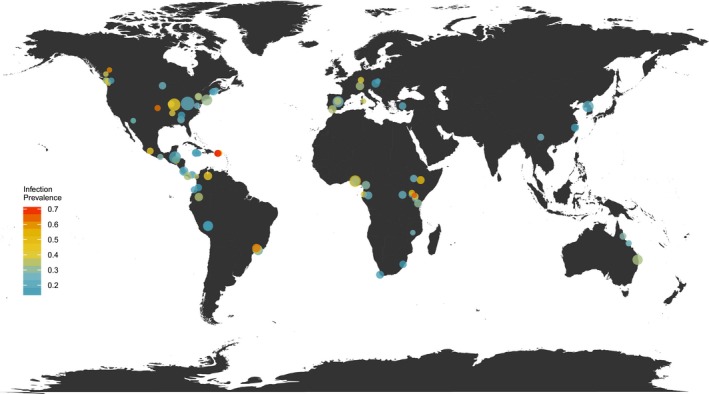
Global map illustrating the 95 amphibian assemblages surveyed for *Batrachochytrium dendrobatidis* from the literature, with each point scaled to the number of species and coloured according to the site‐level infection prevalence

Here, we take advantage of the numerous surveys for *B. dendrobatidis* that have been conducted across the globe to compile a data set of 95 amphibian assemblages representing 302 species that have been exposed to *B. dendrobatidis* (Figure 2). We use this data set to ask how differences in hosts' evolutionary history and traits, while accounting for pathogen and environmental context, contribute to *B. dendrobatidis* infection risk and infection intensity within these assemblages. We further explore whether trait profiles related to tolerance, avoidance or resistance are associated with survival during *B. dendrobatidis* epizootics using our models to predict *B. dendrobatidis* infection risk and intensity for 122 species from five sites in the Neotropics that have experienced *B. dendrobatidis* ‐induced local extinctions and compare that to patterns of species loss. This approach allows us to examine how species traits relate to disease mitigation strategies, and retrospectively ask whether these traits can in turn explain regional patterns of species decline and extinction from *B. dendrobatidis* epizootics.

## 2 METHODS

We followed a multistep analytical approach: analysing global phylogenetic patterns of *B. dendrobatidis* infection risk and intensity, modelling the role of traits, taxonomy, environment and exposure, and subsequently predicting *B. dendrobatidis* infection risk and intensity for species from sites experiencing local extinctions in the Neotropics to evaluate if regional extinction patterns differ among the predicted evolutionary strategies (e.g. tolerance, avoidance, resistance) of hosts towards disease (Figure 1).

### 2.1 *Batrachochytrium dendrobatidis* infection data

We conducted a survey of literature spanning the initial discovery of *B. dendrobatidis* (Berger et al., 1998) up to December 2014, including published journal articles and electronic theses, to identify studies containing information on *B. dendrobatidis* infection prevalence (proportion of individuals infected) across multiple species within a given location. We also considered data from the Global *B. dendrobatidis* ‐Mapping Project (www.bd-maps.net ; Olson et al., 2013), grouping species entries with the same listed sampling date and location as a single site and study for our analysis. We only included *B. dendrobatidis* prevalence data for a species if it had at least five postmetamorphic individuals tested in a given site to ensure a sufficient sampling effort to estimate infection risk and to focus on the postmetamorphic life stages that experience different infection dynamics and experience a much greater fitness cost to infection (Briggs et al., 2010). We only included sites with at least two species present to compare how infection risk varies between species experiencing the same pathogen strain and environmental conditions. We also only included sites with a minimum total infection prevalence of 10% to prevent zero inflation due to low exposure. Only PCR‐based assays of *B. dendrobatidis* infection were considered for consistency. Finally, sampling had to have occurred over the same sampling period (as determined by the author—e.g., those from different sample seasons or years were excluded) for all species within a site to control for the potential variation in pathogen strains and environmental conditions that could confound comparisons between hosts. The vast majority of studies were in systems where *B. dendrobatidis* infections are presumed to be endemic due to a lack of mass mortality at the specific time point of sampling, although in many cases epizootic events may have occurred before or after *B. dendrobatidis* sampling occurred. When estimates of zoospore loads, measured in genomic equivalents, were available from tested individuals, we also retained this information to model infection intensity.

Our literature search yielded a total of 47 studies that met our criteria. This data set (infection prevalence) represents a total of 20,512 swabbed individuals from 504 species‐by‐site observations (median = 18; range = 5–814 per species by site). In total, this data set covers 302 amphibian species from 42 families and includes 95 sites located across seven biogeographic realms (Figure 2). A subset of 19 studies also contained records of zoospore loads from infected individuals (infection intensity), representing 3,381 individuals from 188 species‐by‐site observations (median = 6; range = 1–470), covering 139 species from 33 families in 34 sites.

### 2.2 Phylogenetic patterns

To examine patterns of *B. dendrobatidis* infection in relation to phylogeny, we placed the 302 species on a time‐scaled phylogeny from Pyron and Wiens (2011). Sixty‐six species absent from this phylogeny were placed using close relatives (Table S1). To test if taxonomic affiliations can capture any phylogenetic signal in *B. dendrobatidis* infection patterns, we also created a phylogeny based on taxonomy (Fig. S1).

### 2.3 Trait predictors

We identified 14 biological traits explaining species life history (*N *= 3), climatic niche (*N* = 4), behaviour (*N* = 3) and habitat use (*N *= 4), which may all influence susceptibility to *B. dendrobatidis* infections based on the current literature (Table 1; details in Appendix A). All traits were scored at the species level, as detailed population‐level trait data are unavailable for the vast majority of cases.

**Table 1:** Species‐level trait predictors used to model amphibian *Batrachochytrium dendrobatidis* infection prevalence (*N* = 302 species) and infection intensity (*N *= 139 species), and for assessing disease strategies of amphibians in epizootic sites (*N *= 122 species). The predicted positive (+), negative (−) or neutral (=), associations of each trait to avoidance, resistance and tolerance disease mitigation strategies is indicated

Predictor (units)	Type	Description	Predicted association with each disease strategy		
			Avoidance	Resistance	Tolerance
Body size (mm)	Life history	Mean or mid‐point of male SVL	−	=	+
Clutch size (eggs per clutch)	Life history	Mid‐point of min and max number	=	−	−
Age to sexual Maturity (years)	Life history	Mean time to sexual maturity	−	+	+
Larval habitat (categorical)	Habitat	OP:opportunistic (ponds and streams); PB:ponds; SB:streams; TR:terrestrial	OP,TR: +; PB,SB: −	OP,TR: =; PB,SB: =/+	OP,TR: =; PB,SB: =/+
Adult habitat (categorical)	Habitat	ARB:arboreal; AQ:aquatic; FO:fossorial; TR:terrestrial; SA:semi‐aquatic	ARB, TR: + FO: + AQ, SA: −	ARB, TR: + FO: =/+ AQ, SA: =	ARB, TR: = FO: =/− AQ, SA: =
Habitat breadth (*n*)	Habitat	Number of discrete habitats	+	+	=
Aquatic index (0–1)	Habitat	Proportion of aquatic‐to‐terrestrial habitats.	−	=/+	=/+
Thermal niche position (°C)	Climatic Niche	Mean annual temp across a species geographic range (Bio1)	+	+	=
Thermal niche breadth (°C)	Climatic niche	Mean difference between max and min temp across a species range (Bio7)	+	+	=
Hydric niche position (mm)	Climatic niche	Mean annual precipitation across a species range (Bio12)	−	=/+	=/+
Hydric niche breadth (0‐inf)	Climatic niche	Coefficient of variation in precipitation in a species range (Bio15)	+	+	=
Parental care (factor)	Behaviour	Presence (1) or absence (0) of parental care of young	−	+	=
Migratory (factor)	Behaviour	Presence (1) or absence (0) of migratory behaviour	−	=	=
Breeding system (factor)	Behaviour	Presence of explosive (1) or prolonged (0) breeding systems	−/+	−/+	=

Amphibian natural history still has many gaps, and only 107 species (35%) in our data set had complete coverage for all traits (Table S2). To maximize our coverage to infer trait‐based patterns of *B. dendrobatidis* infection, we imputed missing data for species where we could not collect all traits from the literature. We used two established methods of phylogenetic imputation to estimate missing traits (Penone et al., 2014; Guenard, 2015); the details are in Appendix A. We evaluated the predictive accuracy for each trait using 10‐fold cross‐validation and took the average of five runs for the final imputed traits (Table S3).

### 2.4 Environmental predictors

Temperature and precipitation are known to influence the distribution and prevalence of *B. dendrobatidis* in wild amphibian populations (James et al., 2015; Murray et al., 2011; Nowakowski et al., 2016). We hypothesized that the traits conferring susceptibility to *B. dendrobatidis* infection may differ depending on environmental context. To incorporate trait–environment interactions, we created a climate index for each site (*n *= 95) by extracting 19 temperature and precipitation bioclimatic layers averaged over a 10 km radius around each site (Hijmans, Cameron, Parra, Jones, & Jarvis, 2005). These 19 correlated bioclimatic variables were decomposed into two major principal components that together described 70.2% of the variation. The first principal component loaded positively for warmer, aseasonal and rainy sites (54.0% of variation), and the second principal component loaded positively for annual precipitation and rainfall seasonality (16.2% of variation; Table S4).

### 2.5 Extinction data set

The consequences of *B. dendrobatidis* epizootics have been well documented in the Neotropics, providing an opportunity to test whether traits associated with resistance or tolerance strategies also confer a survival advantage during chytridiomycosis epizootics. For five sites where a known chytridiomycosis epizootic had occurred, we recorded species local persistence or extinction (Catenazzi et al., 2011; Crawford et al., 2010; Lips, 1998, 1999; Woodhams et al., 2008). This data set represented 122 species (20 species also in training data set; mean 30.6 species per site), with species loss ranging from 40% to 78.3% of total richness across sites (mean 55.2%). We collected the same trait and environmental data for each species and site as in the training data set.

### 2.6 Analysis

#### 2.6.1 Phylogenetic signal of *Batrachochytrium dendrobatidis* infections

To first assess whether there was evidence for biology or evolutionary history affecting *B. dendrobatidis* infection prevalence and logarithmic zoospore load, we estimated Pagel's λ using the package MCMCglmm (Hadfield, 2010), which allowed us to control for site‐level exposure. Models were run for 1 × 10^6^ generations with a burn in of 1 × 10^5^ and a sampling interval of 200. For *B. dendrobatidis* prevalence, a binomial error distribution was used, and for infection intensity, we modelled the logarithmic genomic equivalents of *B. dendrobatidis* zoospores with a Gaussian error distribution. Both models used general uninformative priors. Site, study and phylogeny were all included as random effects. Pagel's λ was calculated as the estimated variance explained by the phylogeny relative to residual variance (Hadfield & Nakagawa, 2010).

#### 2.6.2 Boosted regression trees

Following our phylogenetic analysis, we used the machine learning technique of boosted regression trees (BRTs) to model *B. dendrobatidis* infection prevalence and intensity. We cannot include fine‐grained phylogenetic signal with this method, but we note that taxonomic groupings (Order, Family and Genus) capture much of the signal (see Appendix A). BRTs have many advantages in that they fit numerous simple models, allow for interactions between variables and permit nonlinear responses from predictors (Bland et al., 2015; Elith, Leathwick, & Hastie, 2008). We modelled *B. dendrobatidis* prevalence as the logit‐transformed proportion of infected individuals per species per site with a Gaussian error distribution, and each measure of infection prevalence was weighted by the total number of sampled individuals. For *B. dendrobatidis* infection risk, we used the mean logarithmic genomic equivalents of *B. dendrobatidis* across individuals for each species per site with a Gaussian error distribution and weighted each estimate by the number of infected individuals. We modelled *B. dendrobatidis* infection prevalence and intensity based on 14 functional traits (Table 1), two site‐level environmental descriptors and taxonomic classifications for Family (*N *= 42) and Order (*N *= 3). Body size, clutch size and age at maturity were log‐transformed to improve their distributions, and discrete variables and taxon were coded as dummy variables. To account for differential exposure between sites, we included total site‐level infection prevalence of all species as an offset in the model; each species is modelled as under‐ or over‐infected relative to all species in that assemblage. We optimized the BRTs by varying the learning rate and tree complexity (final model for infection risk: lr = 0.002, tc = 10, bag fraction = 0.5; infection intensity: lr = 0.002, tc = 9, bag fraction = 0.5) and assessed model fit based on the mean residual and cross‐validated deviance. As BRTs have a stochastic model building process, we averaged the variable importance and fitted response for each predictor from five runs with the same tuning parameters.

#### 2.6.3 Patterns of extinction

To determine whether traits associated with *B. dendrobatidis* infection also predispose species to extinction during chytridiomycosis epizootics, we used our models to predict *B. dendrobatidis* infection risk and intensity for species in five Neotropical amphibian communities that experienced epizootic extinction events. We averaged the predicted output from five BRT models. We tested whether avoidance, resistance or tolerance strategies were associated with a greater survival probability from chytridiomycosis epizootics by separating species into different strategies based on their predicted infection risk and intensity. We delineated the different evolutionary strategies for disease mitigation (e.g. avoidance, resistance, resistance/avoidance or tolerance, Figure 1) in our predicted data set, based on relative *B. dendrobatidis* infection risk and intensity across species, by creating quadrants using the median predicted infection risk and median predicted infection intensity and assigning populations to each quadrant. To verify the accuracy of this method, we identified eight species from the literature that are known to be susceptible, resistant or tolerant to *B. dendrobatidis* and estimated their predicted infection patterns and subsequent disease mitigation strategy, which performed reasonably well in discriminating species (see Table S7; Fig. S5). We conducted a chi‐square test to determine if the proportion of species that went extinct in each quadrant, representing these different evolutionary strategies, differed from what would be expected by chance. We also conducted tests at the site‐level, which were qualitatively the same (Fig. S6). All analyses were conducted in R v3.3.2.

## 3 RESULTS

### 3.1 Phylogenetic patterns of *Batrachochytrium dendrobatidis* infection

Across 302 species of amphibians, we found that there was a moderate level of phylogenetic signal in *B. dendrobatidis* infection prevalence (Pagel's λ = 0.516, 95% highest posterior density intervals: 0.37, 0.59; Fig. S2), even when accounting for shared geography. For infection intensity, there was a low level of phylogenetic signal in the logarithmic genomic equivalents of zoospores across 139 species (Pagel's λ = 0.216, 95% HPD: 0.12, 0.33; Fig. S3).

### 3.2 Predictors of *Batrachochytrium dendrobatidis* infection prevalence

**Figure 3:**
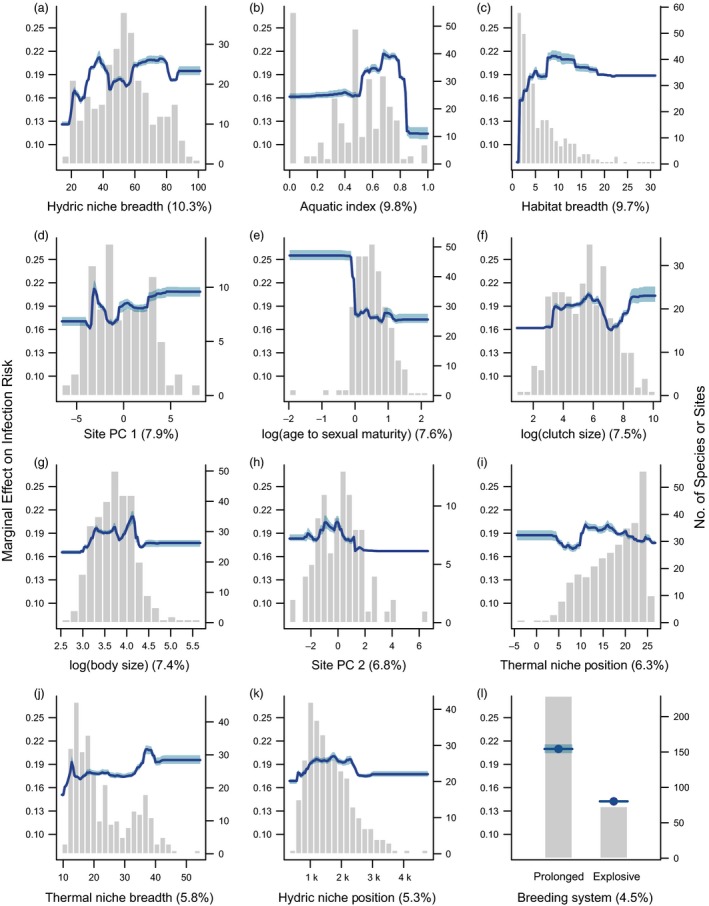
Partial dependence plots illustrating the marginal effect (effect on risk, when all other predictors are averaged) of the top 12 predictors of *Batrachochytrium dendrobatidis* infection prevalence, shown in order of variable importance (indicated by value in parenthesis for each predictor). Background histograms indicate the count distribution of each predictor across the 302 species or 95 sites. Lines indicate the average fitted response across five model runs, and shaded intervals indicate the minimum and maximum fitted response. (a) Hydric niche breadth, (b) Aquatic index, (c) Habitat breadth, (d) Site environment: principle component 1 (see methods for explanation), (e) Age at sexual maturity, (f) Clutch size, (g) Body size, (h) Site environment: principle component 2, (i) Thermal niche position, (j) Thermal niche breadth, (k) Hydric niche position, (l) Breeding system

Variation in *Batrachochytrium dendrobatidis* infection prevalence across amphibians was explained in part by differences in many functional traits (pseudo‐*R* ^2^ = 0.47, range = 0.43–0.50; Figure 3), most with small individual effects (Figure 3). In order of importance (Table S5), species from climates with high rainfall seasonality (i.e., broad hydric niche breadth) had higher infection risk across sites (high: 19%–21%, low: 11%–12%; Figure 3a). Amphibians with a greater association with aquatic habitats (aquatic index) had greater risk of *B. dendrobatidis* infection (19%–22%), although fully aquatic species had lower risk (10.6%; Figure 3b). Contrary to our prediction, species that are highly specialized to one habitat had a lower risk of infection (8%) than species with broader habitat associations (18%–21%; Figure 3c).

Considering life history, small‐bodied species (<20 mm SVL) had a slightly lower risk of infection (16%–17% vs. 18%–20%; Figure 3g), while those species with a rapid sexual maturation (<1 year) had a higher risk of infection (22%–26% vs. 17%–18%). Species with small clutches, fewer than 20 eggs per clutch (risk: 16.6%) and moderately large clutches (1,000–2,500 eggs; risk = 16%) had lower infection risk than their counterparts (17%–20%; Figure 3e).

Aspects of the site‐level climate and species environmental niches influenced patterns of *B. dendrobatidis* infection. Across the 95 sites, amphibian assemblages experiencing warm, less seasonal temperatures (PC1; Figure 3d) and moderate precipitation (PC2; Figure 3h and k) had higher risk of infection compared to other environments. Amphibians that inhabit cooler thermal environments (~10–16°C) had higher risk of infection than species from warmer climates (18.8%–20.6% vs. 17.5%–18%; Figure 3i). Similar to rainfall, species from thermally variable environments also had higher rates of infection (19%–20.5% vs. 15%–17%; Figure 3j). The only behavioural trait that had a strong effect was breeding system: explosive breeders had a lower risk of *B. dendrobatidis* infection (14.2% vs. 20.6%; Figure 3l).

Despite the moderate phylogenetic signal in *B. dendrobatidis* infection prevalence, factors describing taxonomic groupings contributed little to the trait‐dominated BRT models (Table S5). Species from the families Ranidae and Hyperoliidae had a slightly greater, and Bufonidae and Hylidae a slightly lower, risk of infection, although the weak influence of all taxonomic predictors (2.6%) indicates that the raw phylogenetic signal is largely capturing trait variation (Table S5).

### 3.3 Predictors of *Batrachochytrium dendrobatidis* infection intensity

**Figure 4:**
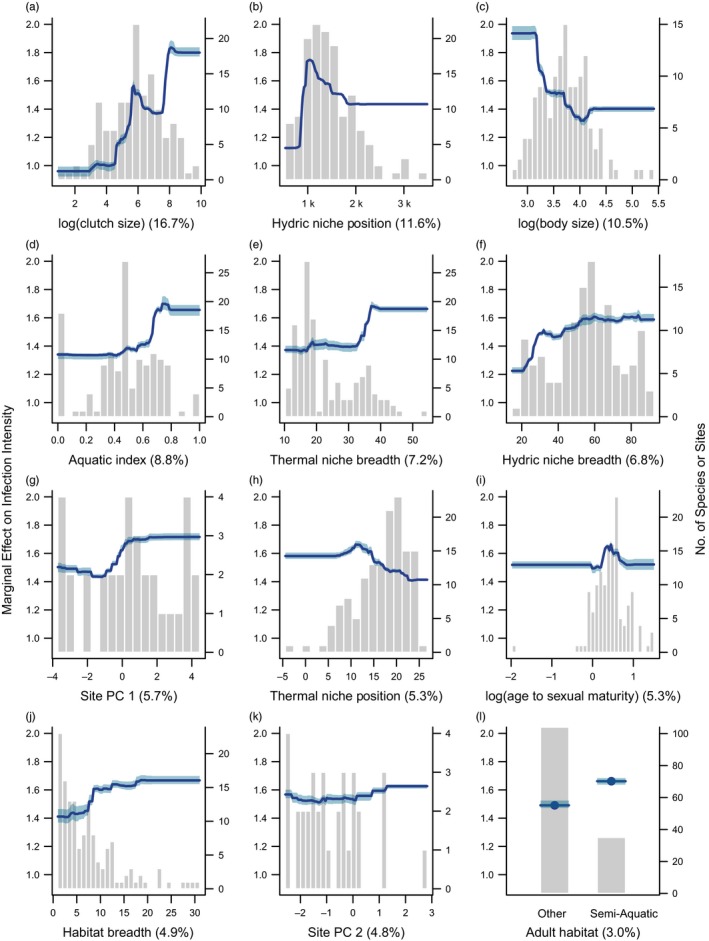
Partial dependence plots illustrating the marginal effect on infection intensity of the top 12 predictors, shown in order of variable importance (indicated by value in parenthesis for each predictor). Background histograms indicate the count distribution of each predictor across the 139 species or 34 sites. Lines indicate the average fitted response across five model runs, and shaded intervals indicate the minimum and maximum fitted response. (a) Clutch size, (b) Hydric niche position, (c) Body size, (d) Aquatic Index, (e) Thermal niche breadth, (f) Hydric niche breadth, (g) Site environment: principle component 1, (h) Thermal niche position, (i) Age at Sexual Maturity, (j) Habitat Breadth, (k) Site environment: principle component 2, (l) Adult habitat: Semi‐aquatic

For the intensity of *B. dendrobatidis* infections (zoospore loads), there was a high degree of variation explained by a combination of traits and site environment (pseudo‐*R* ^2^ = 0.736, range = 0.731–0.741; Figure 4). Species life history appeared to play an important role in patterns of *B. dendrobatidis* infection intensity across infected amphibian assemblages. The most influential predictor of zoospore loads was species clutch size (Table S6), the intensity of infections was higher in highly fecund species and particularly for those with clutch sizes of over 3,000 eggs (Figure 4a). In contrast to patterns of *B. dendrobatidis* infection prevalence, large‐bodied amphibians in infected sites had lower infection intensities compared to small‐bodied species (<35 mm; Figure 4c). Age at sexual maturity was less important in terms of explaining differential infection intensity compared to infection prevalence among species, but those species maturing in 1.5–2.5 years had slightly higher zoospore loads (Figure 4h).

Aspects of species climatic niches were also consistently linked to *B. dendrobatidis* zoospore loads across amphibian assemblages. Rainfall across each species range was associated with infection intensity, where species that live in dry environments (<1,000 mm of precipitation annually) had lower zoospore loads within infected assemblages (Figure 4b). Thermal niche breadth and hydric niche breadth were both positively associated with infection intensity (Figure 4e, f), indicating that species occurring in highly variable environments, in terms of temperature and rainfall, typically had higher levels of infection. Infection intensity was also lower for species that occur in warmer environments (Figure 4i). Site‐level temperature (PC 1) and rainfall (PC2) also influenced infection levels, with higher infection intensity in warmer, less seasonal sites (Figure 4g) and for sites with high levels of precipitation (Figure 4k).

With regard to habitat associations, species that had a greater association with aquatic habitats had higher *B. dendrobatidis* zoospore loads relative to more terrestrial species (Figure 4d), and species with semi‐aquatic adult habitats had an overall greater intensity of infection (Figure 4l). Similar to *B. dendrobatidis* infection risk, habitat specialists actually had a lower intensity of infections than their generalist counterparts (Figure 4j).

Taxonomy and behavioural traits both contributed little to explaining patterns of *B. dendrobatidis* infection intensity in these models, with variable importance totalling 2.15% and 5.05%, respectively (Table S6).

### 3.4 Extinction patterns from chytridiomycosis epizootics

**Figure 5:**
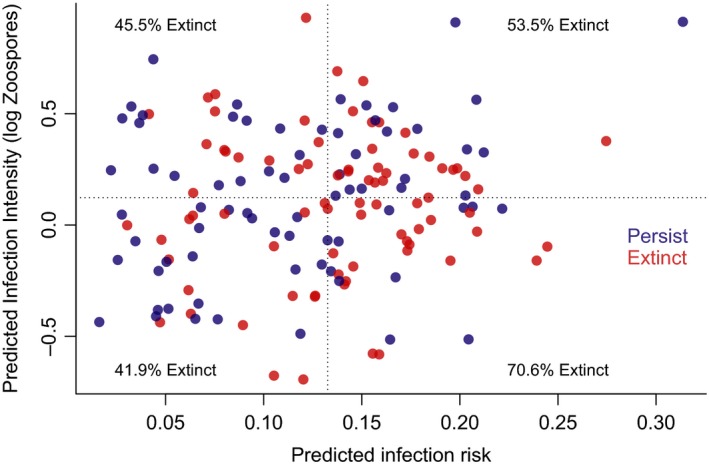
Predicted *Batrachochytrium dendrobatidis* infection risk and intensity for 153 populations of 122 species from five sites experiencing chytridiomycosis epizootics in the Neotropics. Blue points indicate populations that persisted through epizootics, while red points indicate populations that went locally extinct. Populations were assigned to a quadrant of the plot based on median risk and intensity to delineate tolerant, resistant, avoidant or resistant/avoidant strategies. There was no significant difference in the proportion of surviving populations across these quadrants (*Χ* ^*2*^ **= 7.08, *df *= 3, *p *= .069)

When we predicted the relative infection risk and intensity for species from sites experiencing chytridiomycosis epizootics in the Neotropics, we found that there was no clear link between predicted disease mitigation strategy and local extinction outcome (Figure 5). There was a trend for species predicted to be in the quadrant of high infection risk and low intensity, matching a “resistant” strategy, to be more likely to go extinct (Figure 5), but this pattern did not deviate significantly from our null expectation at α = 0.05 (*Χ* ^*2*^ **= 7.08, *df* = 3, *p *= .069).

## 4 DISCUSSION

Across a broad diversity of sites and species, we found consistent patterns of *B. dendrobatidis* infection risk and intensity both with evolutionary history and across a diverse set of species life history and ecological traits. Together with local environment, and accounting for exposure, these components can explain nearly half the variation in *B. dendrobatidis* prevalence (pseudo‐*R* ^2^ = 0.47) and over three quarters of the variation in *B. dendrobatidis* infection intensity (pseudo‐*R* ^2^ = 0.736). Using these global models to predict whether the amphibian species that persist through chytridiomycosis epizootics in the Neotropics exhibited tolerant, resistant or avoidance trait profiles, we found no clear patterns indicating that extinction risk differed across these inferred host disease strategies. This could indicate that the models are unable to accurately identify these different disease mitigation strategies, that these strategies are unrelated to extinction risk from chytridiomycosis epizootics or that other factors may ultimately be more important than these strategies in dictating population persistence during these events.

The functional traits that influenced either risk or intensity of *B. dendrobatidis* infection were broadly consistent with prior studies that have focused on habitat, life history and geographic associations (Bancroft et al., 2011; Bielby et al., 2008; Lips et al., 2003; Murray & Skerratt, 2012), but importantly our results highlight the relative importance of these traits for explaining patterns of *B. dendrobatidis* infection. Species climatic niches, which may reflect differences in species physiology, typically had high variable importance in both *B. dendrobatidis* infection risk and intensity models. Species from highly seasonal environments, in terms of both rainfall and temperature, had both a higher risk of *B. dendrobatidis* infection and greater zoospore loads. This indicates that amphibians from highly seasonal environments may tend towards tolerance phenotypes, a strategy that may be beneficial to cope with high pathogen transmission during seasonal aggregations for breeding (Lenker, Savage, Becker, Rodriguez, & Zamudio, 2014). Thermal physiology has been linked with infection risk (Catenazzi et al., 2014; Nowakowski et al., 2016), but here we show that species water economy may also be key, as both precipitation seasonality and total rainfall were important for *B. dendrobatidis* infection risk and intensity. Species adapted to perennially dry environments may be able to avoid *B. dendrobatidis* zoospores by selecting drier environments (Zumbado‐Ulate, Bolaños, Gutiérrez‐Espeleta, & Puschendorf, 2014), or tolerance to desiccation may provide a means to reduce zoospore loads (Terrell, Engbrecht, Pessier, & Lannoo, 2014). This indicates that species thermal physiology and water economy may directly influence host resistance and tolerance to *B. dendrobatidis*, but challenge experiments with controlled laboratory exposures will be needed to clarify these relationships in a comparative framework (Gahl et al., 2012; Searle et al., 2011).

Amphibian life history and habitat preferences were also important for both infection risk and intensity. Species with high productivity and rapid maturation had a greater risk of *B. dendrobatidis* infection, possibly due to trade‐offs between investment in growth and reproduction versus pathogen defence mechanisms (Han et al., 2015; Johnson et al., 2012). Certain life history traits, such as body size, were associated with a higher infection risk but a lower infection intensity indicating that large‐bodied species, such as bullfrogs (Eskew et al., 2015), may generally exhibit a higher resistance towards *B. dendrobatidis* infection. However, it is interesting to note that for the most part these same large‐bodied, and potentially resistant, species also appeared to be more likely to decline during epizootics in the Neotropics (Lips et al., 2003). This coincides with our result where species exhibiting a phenotype correlated with infection patterns consistent with resistance had the highest proportion of declining populations, and therefore, resistance may not lower extinction risk during epizootics. Habitat associations indicate that both *B. dendrobatidis* infection risk and intensity increase with both habitat generalism and a greater dependence on aquatic habitats (as inferred through the aquatic index), coincident with other studies indicating aquatic species are more susceptible to *B. dendrobatidis* (Bielby et al., 2008; Lips et al., 2003). Whether this indicates a tolerance phenotype for these aquatic, generalist species, or simply a much higher exposure to zoospores, needs additional investigation. These trait associations with *B. dendrobatidis* infection identified in our models have implications for managing amphibians in sites where *B. dendrobatidis* is endemic. In particular, species fitting a high *B. dendrobatidis* risk trait profile may be expected to experience heightened mortality and persistent fitness consequences (Chatfield et al., 2013; Pilliod et al., 2010). Similarly, these traits can potentially be used to identify species that pose a *B. dendrobatidis* biosecurity threat as targets for monitoring or control of human‐mediated transport (Kriger & Hero, 2009). In terms of identifying species susceptible to extinction from *B. dendrobatidis* epizootics, however, these traits appear unable to differentiate resilient and susceptible amphibian species.

Despite the associations between species traits and patterns of *B. dendrobatidis* infection, we found no link between the traits our models identified as driving variation in *B. dendrobatidis* infections with the population persistence of amphibians in chytridiomycosis epizootics in the Neotropics. The heightened transmission of *B. dendrobatidis* infections during epizootic events (Briggs et al., 2010), or the exceptional virulence of introduced *B. dendrobatidis* strains (Piovia‐Scott et al., 2015), may mean that many of the avoidance traits influencing infection risk may simply become irrelevant under epizootic conditions because few species are able to escape infection. Even tolerant or resistant species likely have limits to infection intensity that may be exceeded under epizootic conditions (Bielby, Fisher, Clare, Rosa, & Garner, 2015; Briggs et al., 2010), leading to subsequent decline. Indeed, our results suggest that resistant phenotype species may be the most at risk of extinction during epizootic events, and this may be due to the fitness costs of resisting infection when pathogen encounter rates are high. However, as these disease mitigation strategies can be achieved through multiple mechanisms (i.e., antimicrobial peptides, microbiome, immune systems), it is possible that species exhibiting these different mechanisms have alternative risks of decline during epizootics. We may be obscuring these fine‐scale mechanistic differences by grouping together species that achieve tolerance or resistance through various pathways, and therefore comparing the response to *B. dendrobatidis* of resistant and tolerant species that invoke different underlying mechanisms would be an important future study. If species biology is unrelated to epizootic extinction risk, we would expect random patterns of extinction with respect to evolutionary history. However, an additional analysis of our testing data set indicates a moderately high phylogenetic signal in extinction (Pagel's λ = 0.52; see Appendix A), implying that host evolutionary history in these sites partly influences species survival during epizootics. This suggests that a different set of traits, perhaps relating to aspects of population dynamics rather than disease susceptibility (Tobler, Borgula, & Schmidt, 2012), may determine the ultimate population‐level consequences of chytridiomycosis. Similarly, the postepizootic time frame may matter: species that differ in disease mitigation strategies could have differential time frames for population recovery and recolonization of enzootic sites (Knapp et al., 2016; Scheele et al., 2014). Identifying the traits that underlie persistence, and whether different disease strategies (tolerance, resistance, avoidance and their mechanisms) have different levels of extinction risk during epizootics, will be key to clarifying the mechanisms leading to decline from *B. dendrobatidis* outbreaks.

If species traits influence the risk and intensity of *B. dendrobatidis* infections under endemic conditions, but ultimately do not predict susceptibility to local extinction during epizootic events, how then do we proceed to prioritize amphibians for proactive management? There have been several interesting patterns that have emerged since amphibian assemblages were first devastated by epizootic events. There is evidence that a response to selection towards *B. dendrobatidis* tolerance and resistance is occurring in wild amphibian populations (Savage & Zamudio, 2011, 2016) and that acquired immunity from previous exposure to the pathogen can enhance survival (McMahon et al., 2014). Second, there is evidence that pathogenicity of virulent *B. dendrobatidis* strains can potentially decline over time (Refsnider, Poorten, Langhammer, Burrowes, & Rosenblum, 2015), suggesting that both hosts and pathogens may eventually attain some level of equilibrium. Finally, these two points are strengthened by cases where amphibian species previously devastated by chytridiomycosis are recolonizing sites where *B. dendrobatidis* is still endemic (Knapp et al., 2016; Sapsford, Voordouw, Alford, & Schwarzkopf, 2015; Scheele et al., 2014). Together, these lines of evidence suggest that while *B. dendrobatidis* susceptibility is important, other mechanisms may be just as important for ultimately determining species fates in environments where *B. dendrobatidis* is introduced.

If we consider how management could best prepare for *B. dendrobatidis* epizootics, all this suggests we might do well to focus on identifying species that are restricted to environments and habitats with high *B. dendrobatidis* exposure and may not be able to escape initial introductions of virulent *B. dendrobatidis* strains (Murray & Skerratt, 2012). Species with at least some populations located in sites with low potential for *B. dendrobatidis* colonization are more likely to be buffered from global extinction (Puschendorf et al., 2011; Zumbado‐Ulate et al., 2014). Therefore, it may be more effective to shift focus away from species‐level susceptibility and instead towards the overall risk of *B. dendrobatidis* introduction to amphibian assemblages, ensuring that species in at‐risk sites have potential disease refugia within their distribution and in turn the ability to adapt and recolonize over time.

Confronting the modern biodiversity crisis requires innovative tools to inform efficient management decisions. Although both traits and evolutionary history underlie patterns of *B. dendrobatidis* infection in amphibians, these traits may have limited potential to predict which species will be lost during future chytridiomycosis epizootics at local and regional scales. The lack of association between predicted disease strategy and epizootic extinction risk clarifies that a different approach from identifying and focusing efforts on susceptible species may be warranted to prevent global extinctions from *B. dendrobatidis* introductions. Conservation efforts may be most efficient by focusing on species that entirely overlap with environments that favour *B. dendrobatidis* introduction (Murray & Skerratt, 2012; Puschendorf et al., 2011); for other species, we may simply expect that although significant population losses will likely occur, eventually both pathogen and host may be able to achieve coexistence.

## DATA ACCESSIBILITY

The full data sets including disease and trait data analysed for this study are archived in the Dryad Digital Repository: https://doi.org/10.5061/dryad.t54h6

## APPENDIX A Extended methods & results

### *Batrachochytrium dendrobatidis* infection data

We transformed *Batrachochytrium dendrobatidis* prevalence to normalize the data for our final analysis. We applied a logit transformation to the proportion of infected individuals of a species in a given site. Prior to this transformation, we subtracted 0.1 from each count of infected individuals, or conversely added 0.1 if no individuals were infected, to prevent infinity values. For analyses of phylogenetic signal, which can accommodate a binomial error distribution, all counts were left untransformed.

### Trait data

To determine whether patterns of *B. dendrobatidis* infection prevalence and severity relate to aspects of species functional ecology, we identified 14 potential traits that may influence exposure, resistance or tolerance to *B. dendrobatidis* infections based on empirical evidence in the literature. These traits collectively represent aspects of species life history (3), habitat preferences (4), behaviour (3) and bioclimatic niche (4).

#### Life history

Body size may influence *B. dendrobatidis* infection through several mechanisms: (i) larger amphibians have a greater skin surface area to facilitate infection and carry greater pathogen burdens, (ii) larger amphibians may have greater home ranges that increase chance of exposure to *B. dendrobatidis*. We collected data on mean body size (measured in mm) from various literature sources, using data on males as this had greater coverage across species.

Clutch size can similarly have two effects: (i) high densities of offspring may increase *B. dendrobatidis* infection among tadpoles and subsequently to adults/metamorphs due to the nature of density‐dependent transmission, and (ii) the trade‐off between somatic and gonadic energy investment may result in lower capacities for highly fecund species to resist and tolerate infections (Roy & Kirchner, 2000). We used the mean or mid‐point of the range of clutch sizes (eggs per clutch) reported for a species from the literature.

Age at sexual maturity is a proxy measure of pace of life along the slow‐to‐fast continuum. Species on the slow‐continuum may have greater energetic investment towards resistance and tolerance mechanisms to disease as this would maximize fitness for long‐lived individuals (Johnson et al., 2012; Roy & Kirchner, 2000). Data on the earliest age to sexual maturity (in years) was averaged between males and females.

#### Habitat preferences

The habitat of larvae has long been associated with susceptibility to chytridiomycosis, with stream breeding amphibians being at particular risk of decline and infection (Lips et al., 2003; Kriger & Hero, 2007). We categorized a species larval habitat as either: (i) pond breeding, including both temporary and permanent lentic water bodies, (ii) stream breeding, including any flowing, lotic water bodies, (iii) opportunistic breeders, species that use either lentic or lotic habitats to breed, and (iv) terrestrial breeders, including both direct developers that do not require water as well as phytotelm breeders.

Adult habitat can also influence propensity for infection, as species that occupy aquatic habitats may have greater transmission of *B. dendrobatidis*. We categorized species postmetamorphic habitat as either: (i) terrestrial, including species that occupy the leaf litter or low‐lying vegetation (<2m), (ii) arboreal, species that occupy the upper and mid‐canopy vegetation, (iii) fossorial, species that spend the majority of time underground, (iv) semi‐aquatic, species that typically live in or around aquatic habitats but typically leave the water and are not fully aquatic, (v) aquatic, species that spend the majority of their adult life submerged in lotic or lentic habitats.

The breadth of habitats a species can occupy may influence risk of *B. dendrobatidis* infection by allowing individuals to preferentially avoid areas of high exposure or allowing them to tolerate habitats that may aid in clearing infections (i.e., hot and dry areas). We assessed the habitat breadth of species using the IUCN Habitat Classification Scheme v. 3.1 (IUCN, 2015), counting the number of discrete terrestrial and aquatic habitats that were listed as ‘Suitable’ for a given species.

Given that *B. dendrobatidis* transmission typically occurs from motile zoospores in water, species that are primarily restricted to aquatic habitats may be expected to be at a heightened risk of chytridiomycosis. We calculated an aquatic index for each species, ranging from 0 to 1. This aquatic index was based on the proportion of suitable aquatic‐to‐terrestrial habitats a species occurs in using the aforementioned IUCN Habitat Scheme.

#### Behaviour

Parental care has been theorized to potentially confer benefits to offspring via the vertical transfer of symbiotic microbiota that may inhibit *B. dendrobatidis* infection (Harris et al., 2006). Conversely, parental care may increase *B. dendrobatidis* infections by transmitting zoospores from parent to offspring and vice versa, if there is contact between nonembryonic stages and their parents. We searched for evidence of any parental care (paternal or maternal) reported in the literature for each species, noting its presence or absence.

Migratory behaviour may facilitate the transmission and introduction of *B. dendrobatidis* from spatially disjunct habitats. We assessed migratory behaviour based on reports of reproductive biology in the literature, recording whether species have spatially disjunct breeding and nonbreeding habitats.

Amphibians vary widely in their reproductive mode, but most can be divided into two breeding systems: prolonged and explosive breeders (Wells, 1977). Explosive breeders, which gather in large congregations for short reproductive bouts, may have higher *B. dendrobatidis* transmission among individuals given the high spatio‐temporal overlap of breeding individuals. Conversely, prolonged breeders may be at higher risk of *B. dendrobatidis* infection given that they spend long periods in aquatic breeding habitats, extending the time frame for infection. We categorized explosive and prolonged breeders based on descriptions in the literature, with explosive breeders defined as those that congregate in high densities for short periods (i.e., a few days to weeks).

#### Bioclimatic niche

Species physiology is known to affect *B. dendrobatidis* infections, as individuals can avoid *B. dendrobatidis* by selecting microhabitats outside the fungus thermal growth range or clear infections by raising body temperatures (Catenazzi et al., 2014; Nowakowski et al., 2016; Rowley & Alford, 2013). Unfortunately, the physiological preferences and tolerances of most species are unknown, but macroecological proxies for these traits can be calculated based on the climatic profiles of species distributions and these may reflect broad species‐level differences in physiology (Duarte et al., 2012). To characterize the bioclimatic niche of species, we overlaid spatial polygons of each species extent of occurrence from the IUCN (2015) with bioclimatic layers from WorldClim v1.4 at a resolution of 2.5 min of a latitudinal/longitudinal degree (Hijmans et al., 2005). We calculated measures of thermal preference and niche breadth based on the annual mean temperature (Bio1) and the standard deviation in annual temperature (Bio4) averaged across each species extent of occurrence. We subsequently divided these estimates by 10 to ease interpretation, as their original units are °C × 10. We also used two proxy measures of hydric preference and niche breadth using the annual precipitation across a species range (Bio12) and the coefficient of variation in precipitation, rainfall seasonality (Bio15), averaged across each species range. We assume that these macroecological measures approximate the microhabitat preferences and physiological tolerances of species at the sites where they were examined for *B. dendrobatidis* infections, although whether the realized niches measured at the scale of a species geographic range actually reflect its physiological limits is still uncertain (Duarte et al., 2012; Gouveia et al., 2014).

An overview of trait coverage, and range, across both data sets can be found in Table S2.

### Phylogenetic pairing for data‐poor species

We could not place 66 of the 302 species in our data set on the phylogeny of Pyron and Wiens (2011). Rather than dropping these species from our data set, we opted to get an estimate of their phylogenetic position using surrogate sisters species that we identified based on taxonomy (i.e., congeners) and shared geography (i.e., overlapping ranges or endemism to particular regions). This was done on a species‐by‐species basis, using the best available knowledge for each species. Given the large distances between species in our data set (i.e., hundreds of millions of years) and that the phylogenetic signals in *B. dendrobatidis* infection were robust and very similar at both generic and family levels, we do not expect that using surrogates greatly influenced our results and interpretation.

### Phylogenetic imputation of traits

To maximize our ability to understand trait‐based patterns of *B. dendrobatidis* infection, we opted to impute missing data for species where we could not collect all of the aforementioned traits from the literature. To do this, we used two methods of phylogenetic imputation to estimate the character state (for categorical) or trait value (for continuous) for data‐poor species. The proportion of data missing for each trait varied greatly (see Table S3). with body size having the greatest coverage. Given the widely accepted allometric relationship between clutch size and body size (Duellman & Trueb, 1994) we utilized phylogenetic eigenvector maps with logarithmic body size as a covariate to estimate clutch size using the R package “MPSEM” (Guenard 2015). All species were placed on the time‐scaled phylogeny in Pyron and Wiens (2011). For all other traits, where the inter‐relationships between different traits were not necessarily clear, we utilized a decision tree approach to simultaneously estimate missing character states using the R package “missForest” (Stekhoven, 2013). We utilized an established and effective method that included phylogenetic eigenvectors as fixed effects to estimate traits (Penone et al. 2014), sequentially testing the number of eigenvectors to retain that would maximize predictive accuracy. Across all traits, we found that including 50 phylogenetic eigenvectors as predictors maximized the predictive accuracy. Phylogenetic eigenvectors for the random forest analysis were extracted from the phylogenetic covariance matrix using the “PVRdecomp” function in the R package “PVR” (Santos, Diniz‐Filho, Rangel, & Bini, 2013). In both cases, we evaluated the predictive accuracy (p^2^) as a function of the normalized root mean square error:

$$p^{2}=1-\sqrt{\frac{\text{mean}(\hat{y}-y)}{\text{variance}(y)}}$$

where *y* is the observed trait value and $\hat{y}$ is the value predicted by the model. Perfect estimation of a trait would be indicated by p^2^ = 1. For categorical traits, we estimated the perfect false classified (PFC), a measure of the proportion of times a categorical variable was misclassified. We calculated p^2^ or PFC for each trait using 10‐fold cross‐validation. In all cases, predictive accuracy was high; trait coverage and predictive accuracy are detailed in Table S3.

### Phylogenetic signal analyses

To test if taxonomy served as an appropriate substitute for phylogenetic signal, we conducted our phylogenetic signal analyses on *B. dendrobatidis* infection prevalence and intensity using a taxonomy‐based tree (Fig. S1). Models were run for 1 × 10^6^ generations with a burn in of 1 × 10^5^ and a sampling interval of 200.

The phylogenetic signal in *B. dendrobatidis* infection prevalence based on a taxonomic tree was 0.542 (95% HPD: 0.35, 0.46), in comparison with an estimate of 0.516 (95% HPD: 0.37, 0.49) using the phylogeny of Pyron & Wiens, 2011. For *B. dendrobatidis* infection intensity, the phylogenetic signal using a taxonomic tree (Pagel's λ = 0.219; 95% HPD = 0.08, 0.23) was highly comparable to the real phylogeny (Pagel's λ = 0.187; 95% HPD: 0.11, 0.34). This indicates that the taxonomy captures a similar proportion of the variance in *B. dendrobatidis* prevalence and intensity compared to the phylogeny.

To determine if there were phylogenetic patterns in local extinctions caused by *B. dendrobatidis* epizootics across sites in the Neotropics, we conducted a test of phylogenetic signal on species persistence in our testing data set. Following our analysis of phylogenetic signal in *B. dendrobatidis* infection, we estimated Pagel's λ with a phylogenetic generalized linear model using MCMCglmm (Hadfield, 2010). Species persistence (1) or extinction (0) was modelled as a binomial response, and we included random effects for site (*N* = 5) and the phylogenetic covariance matrix. Models were run for 2.1 × 10^7^ generations with a burn in of 1 × 10^5^ and a sampling interval of 20,000, using uninformative priors. We estimated Pagel's λ as the proportion of variance explained by the phylogenetic covariance matrix, based on the mode of the posterior distribution, relative to the total variance (sum of phylogenetic, site and residual variance).

The estimated contribution of evolutionary history relative to the residual variation in location extinction (equivalent to Pagel's lambda) was 0.516, with 95% highest posterior density intervals of 0.27 (lower) and 0.82 (upper) (Fig. S4). This is accounting for site‐level variation.

### Predicted disease strategies

To verify if our predicted disease mitigation strategies, based on predicted infection risk and intensity, were relatively accurate, we identified a subset of species in our dataset that have evidence of avoidance, resistance or tolerance to *B. dendrobatidis*. The results of this are shown in Table S7 and Fig. S5 and indicate that the models perform adequately in assigning species to their respective categories.
