# Supplementary material for: Amphibian species traits, evolutionary history and environment predict Batrachochytrium dendrobatidis infection patterns, but not extinction risk
Source: Evol Appl. 2017 Sep 3;10(10):1130–45. doi: 10.1111/eva.12520 (PMC5680631; doi:10.1111/eva.12520)
Supplement: Supplementary file 1 [file EVA-10-1130-s001.docx]

**Appendix B – Additional results and figures**

**Table S1 –** Species added to phylogeny according to taxonomy & geography, or as polytomies.

| **Species in dataset** | **Surrogate/Sister species on Pyron & Wiens (2011)** |
| --- | --- |
| *Adenomera marmoratus* | *Adenomera andreae* |
| *Afrixalus clarkei* | *Afrixalus stuhlmanni* |
| *Afrixalus fulvovittatus* | Sister with *Afrixalus laevis* |
| *Afrixalus nigeriensis* | *Afrixalus laevis* |
| *Afrixalus spinifrons* | *Afrixalus knysnae* |
| *Altiphrynoides malcolmi* | *Nectophryne afra* |
| *Ambystoma granulosum* | *Ambystoma mexicanum* |
| *Ambystoma rivulare* | *Ambystoma_andersoni* |
| *Amietia johnstoni* | *Amietia vertebralis* |
| *Amietophrynus funereus* | *Amietophrynus poweri* |
| *Atelopus elegans* | *Atelopus longirostris* |
| *Balebreviceps hillmani* | *Probreviceps macrodactylus* |
| *Bokermannohyla luctuosa* | *Bokermannohyla astartea* |
| *Breviceps sylvestris* | *Breviceps fuscus* |
| *Craugastor chac* | Sister with *Craugastor lineatus* |
| *Craugastor coffeus* | Sister with *Craugastor laticeps* |
| *Craugastor rostralis* | *Craugastor lineatus* |
| *Craugastor sabrinus* | *Craugastor rugulosus* |
| *Crotaphatrema lamottei* | *Crotaphatrema tchabalmbaboensis* |
| *Dendropsophus meridensis* | *Dendropsophus labialis* |
| *Dendropsophus microps* | *Dendropsophus parviceps* |
| *Diasporus gularis* | Sister with *Diasporus diastema* |
| *Eurycea guttolineata* | *Eurycea longicauda* |
| *Flectonotus ohausi* | *Flectonotus fitzgeraldi* |
| *Gastrotheca antoniiochoai* | *Gastrotheca chrysosticta* |
| *Heleophryne rosei* | *Heleophryne purcelli* |
| *Hylodes asper* | *Hylodes ornatus* |
| *Hylodes magalhaesi* | *Hylodes sazimai* |
| *Hyloscirtus alytolylax* | *Hyloscirtus palmeri* |
| *Hyloxalus abditaurantius* | *Hyloxalus pulcherrimus* |
| *Hyperolius burtoni* | *Hyperolius fusciventris* |
| *Hyperolius pickersgilli* | *Hyperolius semidiscus* |
| *Hyperolius sylvaticus* | *Hyperolius chlorosteus* |
| *Idiocranium russeli* | *Grandisonia larvata* |
| *Kaloula borealis* | *Kaloula conjuncta* |
| *Leptopelis aubryi* | Sister with *Leptopelis brevirostris* |
| *Leptopelis christyi* | *Leptopelis bocagii* |
| *Leptopelis crystallinoron* | *Leptopelis brevirostris* |
| *Leptopelis gramineus* | *Leptopelis concolor* |
| *Leptopelis hyloides* | *Leptopelis modestus* |
| *Leptopelis ragazzii* | *Leptopelis vermiculatus* |
| *Leptopelis spiritusnoctis* | Sister with *Leptopelis modestus* |
| *Leptopelis vannutellii* | *Leptopelis argenteus* |
| *Leptopelis xenodactylus* | *Leptopelis natalensis* |
| *Pelophylax caralitanus* | *Pelophylax bedriagae* |
| *Pelophylax chosenicus* | Sister with *Pelophylax nigromaculatus* |
| *Phrynobatrachus liberiensis* | *Phrynobatrachus natalensis* |
| *Phrynobatrachus minutus* | *Phrynobatrachus mababiensis* |
| *Phrynobatrachus plicatus* | *Phrynobatrachus auritus* |
| *Phrynobatrachus steindachneri* | *Phrynobatrachus cricogaster* |
| *Phrynobatrachus werneri* | *Phrynobatrachus africanus* |
| *Plectrohyla dasypus* | *Plectrohyla guatemalensis* |
| *Pristimantis aureolineatus* | *Pristimantis diadematus* |
| *Pristimantis gracilis* | *Pristimantis buckleyi* |
| *Pristimantis salaputium* | *Pristimantis ardalonychus* |
| *Pristimantis thectopternus* | *Pristimantis calcarulatus* |
| *Pristimantis waoranii* | *Pristimantis unistrigatus* |
| *Pseudophryne raveni* | *Pseudophryne bibronii* |
| *Psychrophrynella usurpator* | *Psychrophrynella wettsteini* |
| *Ptychadena erlangeri* | Sister with *Ptychadena cooperi* |
| *Ptychadena neumanni* | *Ptychadena cooperi* |
| *Rana coreana* | *Rana amurensis* |
| *Scarthyla vigilans* | *Scarthyla goinorum* |
| *Scinax hayii* | *Scinax crospedospilus* |
| *Taudactylus eungellensis* | *Taudactylus acutirostris* |
| *Uperoleia fusca* | *Uperoleia laevigata* |

**Table S2 –** Overview of the trait distribution and coverage for species in both training and testing datasets.

| Predictor (units) | Range/Count (N) per Dataset | |
| --- | --- | --- |
|  | Training (N = 302) | Testing (N = 122) |
| Body Size (mm) | 12.5-290, N = 302 | 10.7-169, N = 122 |
| Clutch Size (eggs per clutch) | 2.5-25000, N = 230 | 2.5-7500, N = 69 |
| Age at Maturity (years) | 0.139-9, N = 117 | 0.5-2.5, N = 3 |
| Larval Habitat | OP: 72  PB: 109  SB: 59  TR: 63  N = 302 | OP: 7  PB: 9  SB: 42  TR: 62  N = 120 |
| Adult Habitat | AQ: 19  ARB: 87  FO: 15  SA: 73  TR: 107  N= 302 | AQ: 0  ARB: 49  FO: 1  SA: 20  TR: 51  N= 121 |
| Habitat Breadth (n) | 1-31, N = 298 | 1-31, N = 120 |
| Aquatic Index | 0-1, N = 298 | 0-1, N = 120 |
| Thermal Niche Position (˚C) | -4.42-26.7, N = 298 | 8.63-26.2, N = 119 |
| Thermal Niche Breadth (˚C) | 9.61-54.5, N = 298 | 9.63-22.9, N = 119 |
| Hydric Niche Position (mm) | 337.7-4777, N = 298 | 486-5066, N = 119 |
| Hydric Niche Breadth | 14.1-101.2, N = 298 | 28.6-105.3, N = 119 |
| Parental Care | 1: 48  0: 165  N = 213 | 1: 31  0: 32  N = 63 |
| Migratory | 1: 149  0: 73  N = 222 | 1: 32  0: 15  N = 47 |
| Breeding System | 1: 69  0: 153  N = 222 | 1: 4  0: 43  N = 47 |

**Table S3** Missing data and predictive accuracy for traits across both the training (302 species) and testing dataset (105 species), overall 15.34% of traits were missing values.

| Trait | No. Missing Species (%) | Predictive Accuracy (p^2^) or Percent True Classified (PTC) |
| --- | --- | --- |
| Body Size (SVL, mm) | 0 (0%) | NA |
| Clutch Size (# eggs) | 129 (29.3%) | 0.832 |
| Age to Maturity (years) | 309 (70.4%) | 0.224 |
| Larval Habitat | 2 (0.4%) | 68.6% |
| Adult Habitat | 2 (0.03%) | 75.3% |
| Habitat Breadth | 7 (1.6%) | 0.482 |
| Aquatic Index | 7 (1.6%) | 0.606 |
| Parental Care | 154 (35.1%) | 87.4% |
| Migratory | 153 (34.9%) | 82.9% |
| Breeding System | 150 (34.2%) | 82.7% |
| Thermal Niche | 7 (1.6%) | 0.757 |
| Thermal Niche Breadth | 7 (1.6%) | 0.845 |
| Hydric Niche | 7 (1.6%) | 0.801 |
| Hydric Niche Breadth | 7 (1.6%) | 0.493 |

**Table S4 –** Principle component loadings for the 19 bioclimatic descriptors from WordClim (Hijmans et al. 2005), used to describe each sites’ climate index, with major loadings highlighted in bold. Variable descriptions can be found at <http://www.worldclim.org/bioclim>.

| Variable | Principle component 1 | Principle component 2 |
| --- | --- | --- |
| Bio1 | **0.295007** | -0.11907 |
| Bio2 | -0.12952 | -0.19212 |
| Bio3 | **0.251529** | -0.15971 |
| Bio4 | **-0.26228** | 0.176648 |
| Bio5 | 0.140228 | -0.0527 |
| Bio6 | **0.30064** | -0.11565 |
| Bio7 | **-0.27802** | 0.107393 |
| Bio8 | **0.208306** | -0.10432 |
| Bio9 | **0.252838** | -0.14503 |
| Bio10 | **0.204443** | -0.00829 |
| Bio11 | **0.296893** | -0.15038 |
| Bio12 | **0.270333** | **0.251454** |
| Bio13 | **0.275896** | 0.08457 |
| Bio14 | 0.108966 | **0.488672** |
| Bio15 | 0.09183 | -0.43002 |
| Bio16 | **0.275413** | 0.109624 |
| Bio17 | 0.130564 | **0.475982** |
| Bio18 | **0.208751** | 0.175249 |
| Bio19 | 0.183179 | **0.215706** |

**Table S5 –** Relative influence, a measure of how often a predictor is selected and how well it improves the model, for all trait (N = 24), environment (N= 2), and taxonomic predictors (N = 45) explaining variation in *Batrachochytrium dendrobatidis* infection prevalence.

| **Predictor** | **Relative Influence (%)** |
| --- | --- |
| Hydric Niche Breadth | 10.31 |
| Aquatic Index | 9.76 |
| Habitat Breadth | 9.74 |
| Site Environment – PC1 | 7.92 |
| Age at Maturity | 7.62 |
| Clutch Size | 7.54 |
| Body Size | 7.41 |
| Site Environment – PC2 | 7.40 |
| Thermal Niche Position | 6.81 |
| Thermal Niche Breadth | 6.01 |
| Hydric Niche Position | 5.28 |
| Breeding System | 4.46 |
| Adult Habitat: Aquatic | 1.78 |
| Adult Habitat: Semi-aquatic | 1.63 |
| Adult Habitat: Terrestrial | 0.87 |
| Family: Bufonidae | 0.80 |
| Family: Hyperoliidae | 0.66 |
| Larval Habitat: Ponds | 0.61 |
| Larval Habitat: Opportunistic | 0.60 |
| Migratory | 0.49 |
| Larval Habitat: Streams | 0.47 |
| Family: Ranidae | 0.39 |
| Family: Hylidae | 0.32 |
| Order: Anura | 0.28 |
| Adult Habitat: Arboreal | 0.21 |
| Family: Eleutherodactylidae | 0.19 |
| Parental Care | 0.13 |
| Family: Plethodontidae | 0.07 |
| Family: Salamandridae | 0.06 |
| Family: Strabomantidae | 0.06 |
| Order: Caudata | 0.05 |
| Larval Habitat: Terrestrial | 0.03 |
| Adult Habitat: Fossorial | 0.03 |
| Family: Craugastoridae | 0.01 |
| Family: Alytidae | 0.00 |
| Family: Ambystomatidae | 0.00 |
| Family: Amphignathodontidae | 0.00 |
| Family: Arthroleptidae | 0.00 |
| Family: Bombinatoridae | 0.00 |
| Family: Brachycephalidae | 0.00 |
| Family: Breviceptidae | 0.00 |
| Family: Centrolenidae | 0.00 |
| Family: Ceratophryidae | 0.00 |
| Family: Conrauidae | 0.00 |
| Family: Cryptobranchidae | 0.00 |
| Family: Cycloramphidae | 0.00 |
| Family: Dendrobatidae | 0.00 |
| Family: Dermophiidae | 0.00 |
| Family: Dicroglossidae | 0.00 |
| Family: Heleophrynidae | 0.00 |
| Family: Hemiphractidae | 0.00 |
| Family: Hylodidae | 0.00 |
| Family: Hynobiidae | 0.00 |
| Family: Indotyphlidae | 0.00 |
| Family: Leptodactylidae | 0.00 |
| Family: Microhylidae | 0.00 |
| Family: Myobatrachidae | 0.00 |
| Family: Pelobatidae | 0.00 |
| Family: Pelodytidae | 0.00 |
| Family: Petropedetidae | 0.00 |
| Family: Phrynobatrachidae | 0.00 |
| Family: Pipidae | 0.00 |
| Family: Proteidae | 0.00 |
| Family: Ptychadenidae | 0.00 |
| Family: Pyxicephalidae | 0.00 |
| Family: Rhacophoridae | 0.00 |
| Family: Scaphiopodidae | 0.00 |
| Family: Scolecomorphidae | 0.00 |
| Order: Gymnophiona | 0.00 |

**Table S6 –** Relative influence, a measure of how often a predictor is selected and how well it improves the model, averaged across 5 boosted regression tree models for all trait (N = 24), environment (N= 2), and taxonomic predictors (N = 45) explaining variation in log genomic equivalents of *Batrachochytrium dendrobatidis* zoospores.

| **Predictor** | **Relative Influence (%)** |
| --- | --- |
| Clutch Size | 16.67 |
| Hydric Niche Position | 11.60 |
| Body Size | 10.52 |
| Aquatic Index | 8.81 |
| Thermal Niche Breadth | 7.21 |
| Hydric Niche Breadth | 6.75 |
| Site Environment – PC1 | 5.74 |
| Thermal Niche Position | 5.27 |
| Age at Maturity | 5.26 |
| Habitat Breadth | 4.93 |
| Site Environment – PC2 | 4.82 |
| Adult Habitat: Semi-aquatic | 2.98 |
| Family: Hylidae | 2.22 |
| Order: Anura | 1.40 |
| Migratory | 1.16 |
| Breeding System | 0.97 |
| Order: Caudata | 0.94 |
| Adult Habitat: Terrestrial | 0.69 |
| Larval Habitat: Ponds | 0.67 |
| Adult Habitat: Arboreal | 0.62 |
| Family: Ranidae | 0.49 |
| Larval Habitat: Opportunistic | 0.21 |
| Larval Habitat: Streams | 0.03 |
| Parental Care | 0.03 |
| Larval Habitat: Terrestrial | 0.02 |
| Adult Habitat: Aquatic | 0.00 |
| Adult Habitat: Fossorial | 0.00 |
| Family: Plethodontidae | 0.00 |
| Family: Salamandridae | 0.00 |
| Family: Strabomantidae | 0.00 |
| Family: Eleutherodactylidae | 0.00 |
| Family: Bufonidae | 0.00 |
| Family: Hyperoliidae | 0.00 |
| Family: Craugastoridae | 0.00 |
| Family: Alytidae | 0.00 |
| Family: Ambystomatidae | 0.00 |
| Family: Amphignathodontidae | 0.00 |
| Family: Arthroleptidae | 0.00 |
| Family: Bombinatoridae | 0.00 |
| Family: Brachycephalidae | 0.00 |
| Family: Breviceptidae | 0.00 |
| Family: Centrolenidae | 0.00 |
| Family: Ceratophryidae | 0.00 |
| Family: Conrauidae | 0.00 |
| Family: Cryptobranchidae | 0.00 |
| Family: Cycloramphidae | 0.00 |
| Family: Dendrobatidae | 0.00 |
| Family: Dermophiidae | 0.00 |
| Family: Dicroglossidae | 0.00 |
| Family: Heleophrynidae | 0.00 |
| Family: Hemiphractidae | 0.00 |
| Family: Hylodidae | 0.00 |
| Family: Hynobiidae | 0.00 |
| Family: Indotyphlidae | 0.00 |
| Family: Leptodactylidae | 0.00 |
| Family: Microhylidae | 0.00 |
| Family: Myobatrachidae | 0.00 |
| Family: Pelobatidae | 0.00 |
| Family: Pelodytidae | 0.00 |
| Family: Petropedetidae | 0.00 |
| Family: Phrynobatrachidae | 0.00 |
| Family: Pipidae | 0.00 |
| Family: Proteidae | 0.00 |
| Family: Ptychadenidae | 0.00 |
| Family: Pyxicephalidae | 0.00 |
| Family: Rhacophoridae | 0.00 |
| Family: Scaphiopodidae | 0.00 |
| Family: Scolecomorphidae | 0.00 |
| Order: Gymnophiona | 0.00 |

**Table S7 –** Comparison of disease strategy inferred from experimental *Bd* infections and predicted infection risk (IR) and infection load (IL) based on traits correlated with *Bd* infection prevalence and intensity from this study for a subset of species in our testing dataset (Fig. S5).

| **Species** | **Disease strategy** | **Reference** | **Predicted** |
| --- | --- | --- | --- |
| *Atelopus chiriquiensis* | Avoidance (Susceptible) | (La Marca et al. 2005) | Medium IR,  High IL |
| *Atelopus erythropus* | Avoidance (Susceptible) | (La Marca et al. 2005) | Low IR,  High IL |
| *Atelopus varius* | Avoidance (Susceptible) | (La Marca et al. 2005) | Medium IR,  Medium IL |
| *Atelopus zeteki* | Avoidance (Susceptible) | (Ellison et al. 2015) | Medium IR,  Medium IL |
| *Agalychnis callydra* | Resistance | (Ellison et al. 2015) | High IR, Medium IL |
| *Craugastor fitzingeri* | Resistance | (Ellison et al. 2015) | High IR, Medium IL |
| *Gastrotheca excubitor* | Resistance | (Burkart 2015) | Low IR,  Low IL |
| *Rhinella marina* | Resistance | (Poorten and Rosenblum 2016) | Low IR,  High IL |

**
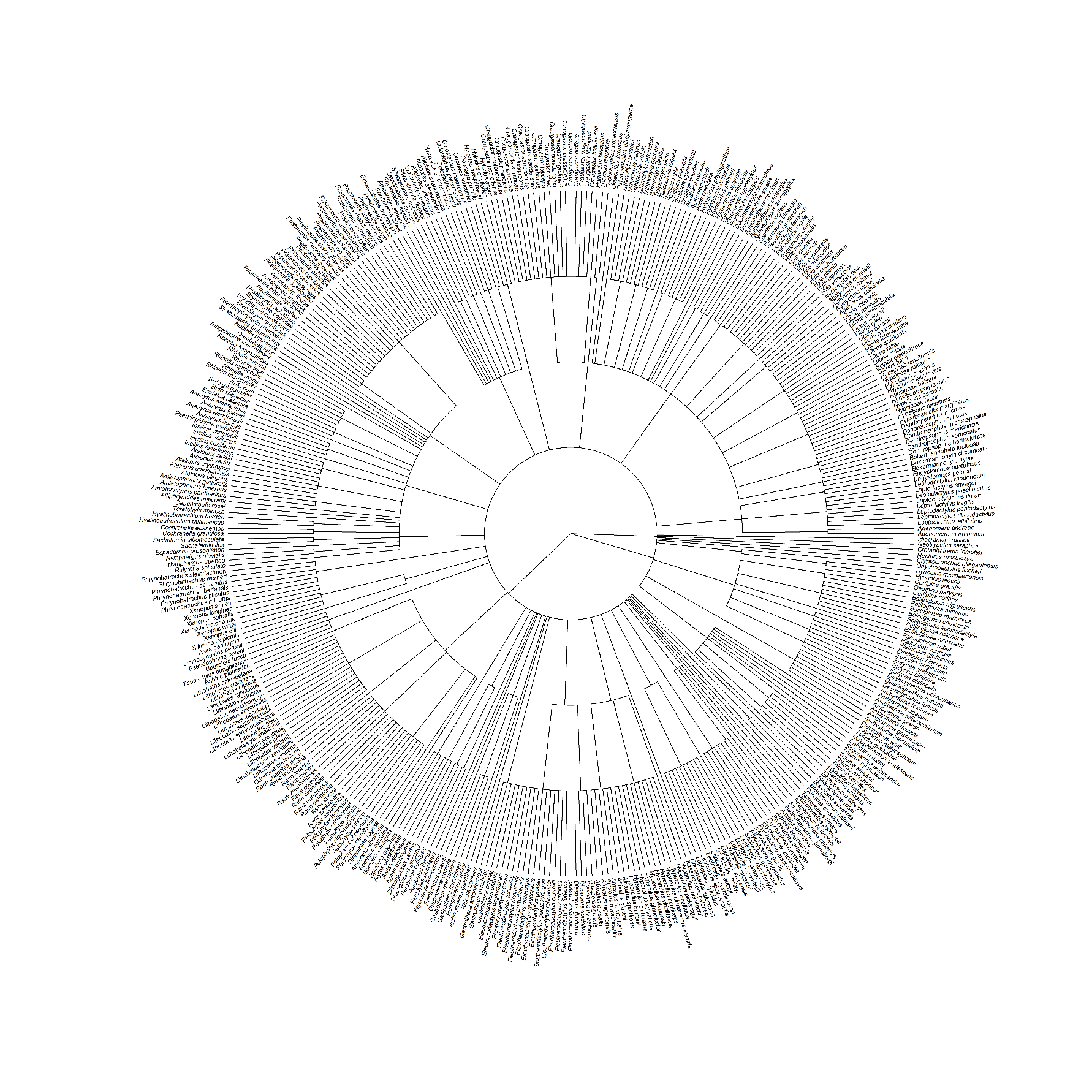
**

**Figure S1.** Phylogeny constructed based on taxonomic relationships to test whether taxonomic groupings can capture the phylogenetic signal in *Bd* infection prevalence and intensity.

**
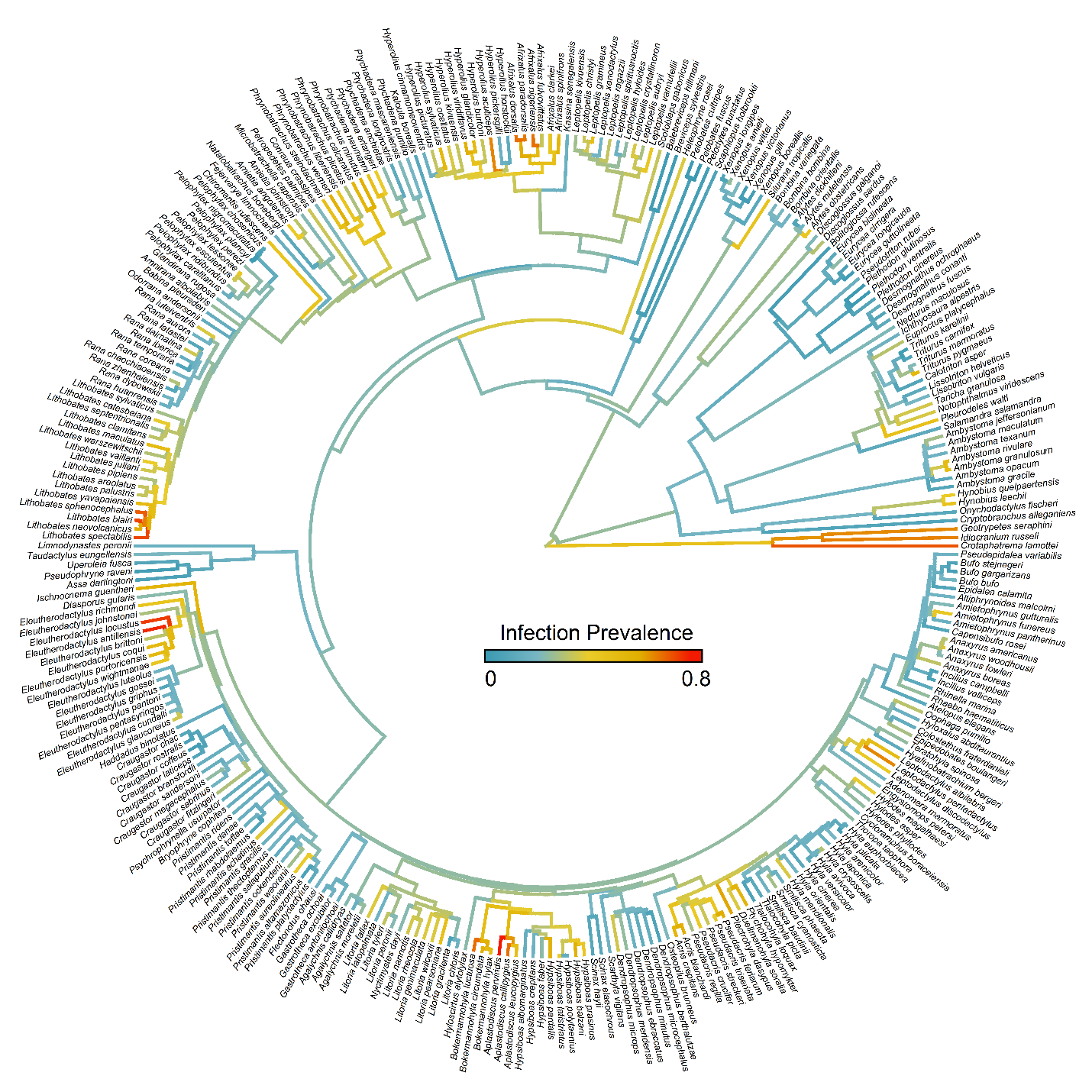
**

**Figure S2**. Phylogeny of the 302 species in the dataset for *Batrachochytrium dendrobatidis* infection prevalence, with branch lengths colour-coded to species’ total infection prevalence summed across sites indicating those with low (blue) to high (red) prevalence. Ancestral states were estimated by maximum likelihood using the ‘fastAnc’ function (Revell 2012).

**
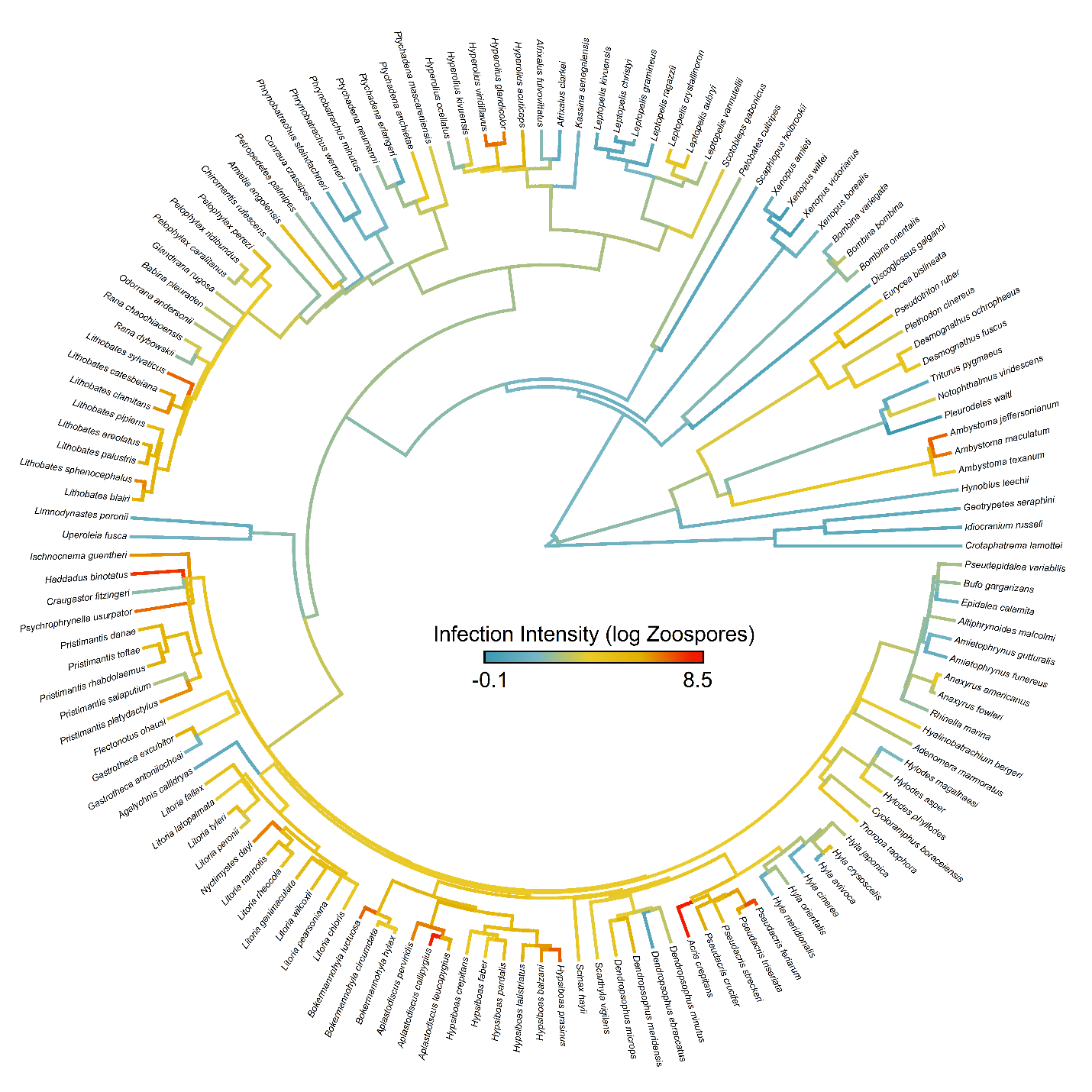
**

**Figure S3**. Phylogeny of the 139 species in the dataset for *Batrachochytrium dendrobatidis* infection intensity, with branch lengths colour-coded to species’ mean logarithmic zoospore load indicating those with low (blue) to high (red) zoospore counts. Ancestral states were estimated by maximum likelihood using the ‘fastAnc’ function (Revell 2012).

**
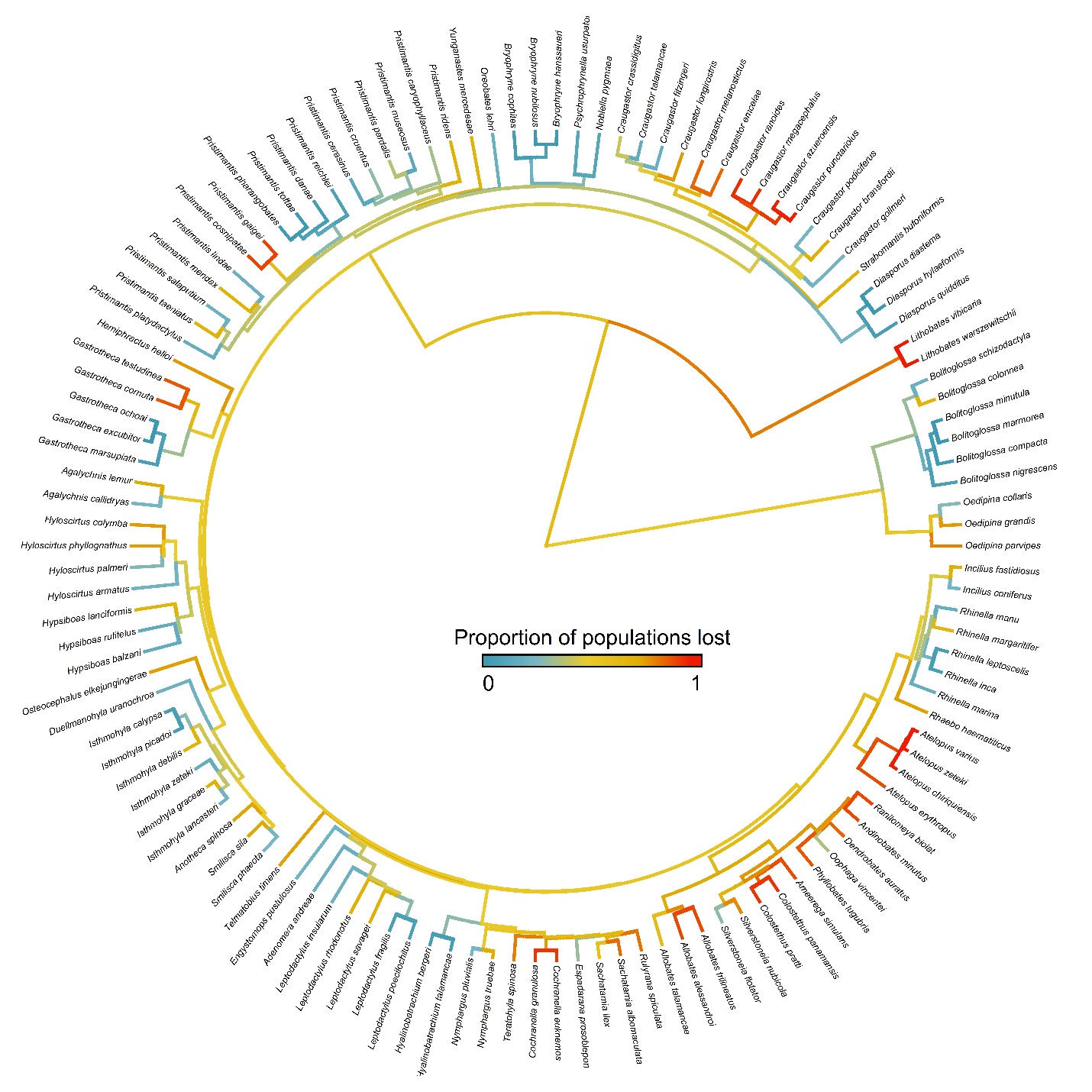
**

**Figure S4**. Phylogeny of 122 species that either persisted or went extinct during chytridiomycosis epizootics in 5 sites from the Neotropics. Branch lengths are colour-coded to the proportion of populations lost during epizootics for each species with ranging from no extinction (blue) to total population loss (red). Ancestral states were estimated by maximum likelihood using the ‘fastAnc’ function (Revell 2012).

**
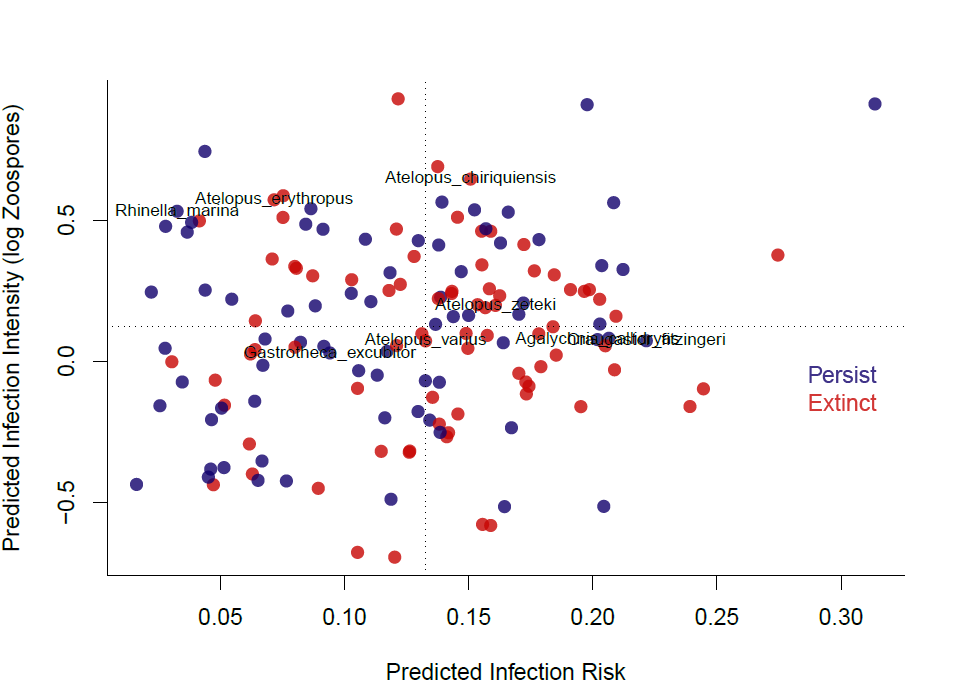
**

**Figure S5**. Predicted disease strategies for species’ known to be susceptible (*Atelopus*), resistant (*G. excubitor*, *A. callidryas*, *R. marina*), or tolerant (*C. fitzingeri*) to *Bd*.

**
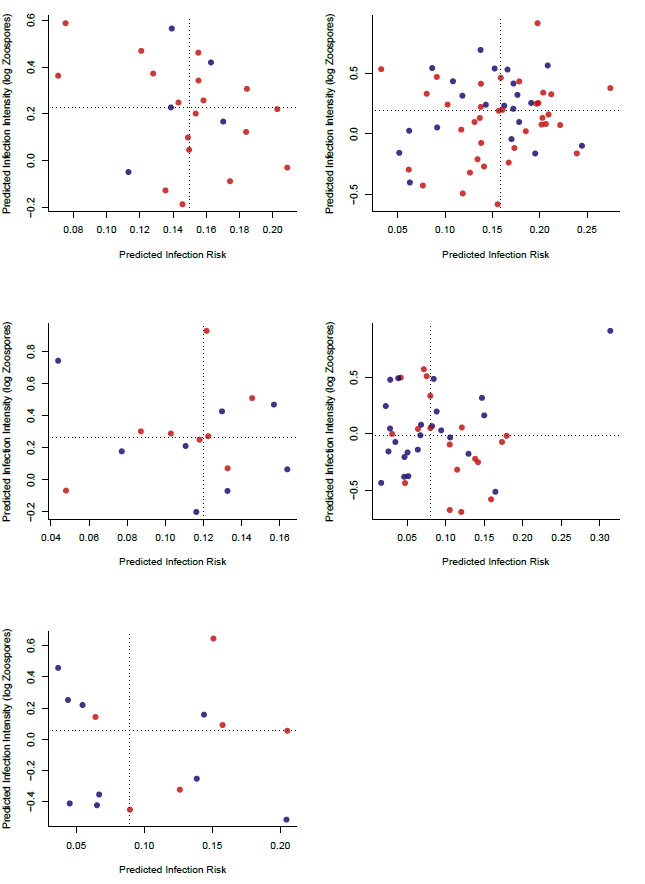
**

**Figure S6.** Site-level plots of predicted infection risk and intensity and their link to persistence (blue) vs. local extinction (red). A) Altos de Campana National Park, Panama, B) El Copé, Panama, C) Reserve Forestal Fortuna, Panama, D) , Kosñipata Valley, Peru E) Las Tablas, Costa Rica (Lips 1998). The only community that showed a significant difference in extinction outcome was in the Kosñipata Valley, Peru (D), Χ^2^ = 11.7, *p* = 0.008; where resistant phenotype species had a higher proportion of extinctions.

**Literature Cited**

Burkart, D. 2015. Understanding chytridiomycosis resistance by investigating the cutaneous defense mechanisms of Marsupial frogs. Sourthern Illinois University Carbondale.

Ellison, A. R., T. Tunstall, G. V DiRenzo, M. C. Hughey, E. A. Rebollar, L. K. Belden, R. N. Harris, R. Ibáñez, K. R. Lips, and K. R. Zamudio. 2015. More than skin deep: functional genomic basis for resistance to amphibian chytridiomycosis. Genome Biol. Evol. 7:286–98. Oxford University Press.

Hijmans, R. J., S. E. Cameron, J. L. Parra, P. G. Jones, and A. Jarvis. 2005. Very high resolution interpolated climate surfaces for global land areas. Int. J. Climatol. 25:1965–1978.

La Marca, E., K. R. Lips, S. Lötters, R. Puschendorf, R. Ibáñez, J. V. Rueda-Almonacid, R. Schulte, C. Marty, F. Castro, J. Manzanilla-Puppo, J. E. Garcia-Pérez, F. Bolaños, G. Chaves, J. A. Pounds, E. Toral, and B. E. Young. 2005. Catastrophic population declines and extinctions in neotropical harlequin frogs (Bufonidae: Atelopus).

Poorten, T. J., and E. B. Rosenblum. 2016. Comparative study of host response to chytridiomycosis in a susceptible and a resistant toad species. Mol. Ecol. 25:5663–5679.

Revell, L. J. 2012. phytools: an R package for phylogenetic comparative biology (and other things). Methods Ecol. Evol. 3:217–223. Blackwell Publishing Ltd.
